# Clinical heterogeneity and treatment outcomes of extrapulmonary tuberculosis in a low-incidence setting: insights from a prospective cohort study

**DOI:** 10.1007/s15010-025-02500-4

**Published:** 2025-03-11

**Authors:** Angela Klingmüller, Marie Feldmann, Samuel Rohr, Lea Helmhold, Lena Junker, Margarete Scherer, Jörg-Janne Vehreschild, Kirsten Schmidt-Hellerau, Ada Hoffmann, Jonathan Jantsch, Alexander Simonis, Victor Suárez, Dominic Rauschning, Natalie Funke, Jakob J. Malin, Lena M. Biehl, Philipp Schommers, Gerd Fätkenheuer, Clara Lehmann, Jan Rybniker, Isabelle Suárez

**Affiliations:** 1 Department I of Internal Medicine, Division of Infectious Diseases, Medical Faculty and University Hospital Cologne, University of Cologne, Kerpener Str. 62, 50937 Cologne, Germany; 2 Center for Molecular Medicine Cologne (CMMC), Medical Faculty and University Hospital Cologne, University of Cologne, 50937 Cologne, Germany; 3 Department of Orthopedics and Trauma surgery, Bundeswehr Central Hospital Koblenz, Koblenz, Germany; 4 German Center for Infection Research (DZIF), Bonn-Cologne, Germany; 5 Faculty of Medicine, Institute for Digital Medicine and Clinical Data Science, Goethe University Frankfurt, Frankfurt, Germany; 6 Institute for Medical Microbiology, Immunology and Hygiene, University Hospital of Cologne, Cologne, Germany; 7 Department IB of Internal Medicine, Bundeswehr Central Hospital Koblenz, Koblenz, Germany; 8 Municipal Health Authority Cologne, Cologne, Germany; 9 Department II of Internal Medicine (Nephrology, Rheumatology, Diabetes, and General Internal Medicine, Center for Molecular Medicine Cologne, Cologne, Germany; 10 Emergency Department, University of Cologne, Cologne, Germany; 11 Department of Internal Medicine, Evangelisches Krankenhaus Düsseldorf, Düsseldorf, Germany

**Keywords:** Extrapulmonary tuberculosis, EPTB, Paradoxical reaction, Treatment response assessment, Disseminated tuberculosis, Lymph node tuberculosis

## Abstract

**Purpose:**

Tuberculosis (TB) remains a leading cause of morbidity and mortality, with 1.3 million deaths in 2022. Extrapulmonary tuberculosis (EPTB) accounts for approximately 20% of all TB cases. We assessed the clinical presentation and challenges during the course of treatment in EPTB patients in a low-incidence setting.

**Methods:**

We conducted a prospective cohort study involving 44 EPTB patients at the University Hospital of Cologne, Germany. Clinical data were collected before and during treatment.

**Results:**

The cohort comprised 44 patients originating from 21 countries. Two or more invasive procedures were required for microbiological confirmation in 59% (26/44) of the cases. Sputum culture was positive in 18% (8/44) of patients, with 63% (5/8) showing no radiological signs of pulmonary involvement. The median therapy duration was ten months and increased with disease severity. Paradoxical reactions (PR) occurred in 31% (13/42) of the patients. A previously published clinical scoring system assessing EPTB treatment responses showed a favorable treatment outcome in only 68% (21/31) of the patients in this cohort.

**Conclusion:**

EPTB exhibits highly variable disease severity and organ involvement. Treatment initiation is often delayed due to diagnostic challenges. Management is complicated by the frequent occurrence of PR, which can lead to treatment durations exceeding standard recommendations. Clinical scores for treatment response assessment may not be reliably applicable, highlighting the need for alternative biomarkers.

**Supplementary Information:**

The online version contains supplementary material available at 10.1007/s15010-025-02500-4.

## Introduction

### Background

In 2022, an estimated 10.6 million new cases of tuberculosis (TB) were reported, resulting in 1.3 million deaths worldwide [1]. While pulmonary involvement is more common globally, extrapulmonary TB (EPTB) accounts for approximately 20% of TB cases worldwide. The World Health Organization (WHO) defines EPTB as TB that is clinically diagnosed or microbiologically confirmed to affect organs other than the lungs [2].

In the EEA (European Economic Area) and (European Union) region, the proportion of EPTB among all TB cases is increasing [3]. However, the rates of EPTB among TB cases vary significantly, ranging from 2.7% in Hungary to 40.8% in the Netherlands. Overall, EPTB is more prevalent in the northern and western regions of the EU [4, 5].

In Germany, EPTB cases increased from 22.6% in 2010 to 28.7% in 2020, with a significant proportion occurring among migrants, while rates decreased to 23.9% in 2022 [5, 6, 7, 8]. This downward trend in EPTB was likely influenced by the arrival of migrants from Ukraine in 2022, who predominantly had PTB, thereby reducing the overall proportion of EPTB cases [9].

Timely diagnosis of EPTB is challenging due to its diverse clinical presentations, which often mimic other diseases. The diagnostic process is further complicated by the insidious and less overt nature of EPTB, often requiring advanced and invasive procedures, such as biopsies and imaging techniques. These factors, along with varying awareness and prevalence across regions, contribute to significant delays in diagnosis and treatment, thereby complicating management. Despite the rising incidence of EPTB in some areas, studies focusing exclusively on EPTB are limited [10]. Although the WHO recommends a 6-month therapy for most forms of EPTB [2], the appropriateness of this standard approach is debatable, as treatment success rates for EPTB patients often fall below the WHO target of ≥ 90% [11]. Consequently, we initiated a prospective observational clinical cohort study with the aim of advancing our comprehension of the clinical presentation, management and treatment of EPTB patients.

### Objectives

The primary goal of this study was to deepen our understanding of the diverse clinical presentations and diagnostic hurdles associated with EPTB. Additionally, the study aimed to correlate treatment durations with the extent of organ involvement, evaluate the prevalence of paradoxical reactions (PR), and validate a clinical scoring system [12] as an indicator of treatment success.

## Methods

### Study participants

Patients undergoing treatment for EPTB at the University Hospital of Cologne, Germany, were enrolled in a prospective, longitudinal cohort study (Cologne EX-TB study). Inclusion criteria encompassed diagnosed EPTB based on the isolation of bacteria from the *Mycobacterium tuberculosis* complex in bodily secretions or tissues using PCR or culture, aligning with the WHO’s definition of ‘bacteriologically confirmed TB’; or a presumptive clinical diagnosis of EPTB consistent with the WHO’s definition of ‘clinically diagnosed TB’ [2]. Patients with concurrent pulmonary and extrapulmonary manifestation were included, if the latter was clinically leading. The recruitment period extended from August 2018 to January 2023. Written informed consent was obtained from each study participant.

### Study design

Study visits were conducted longitudinally at various time points: at the time of diagnosis, referred to as ‘baseline,’ one month and three months after treatment initiation, and every three months during treatment, referred to as ‘follow-up visits’. At baseline, descriptive patient data were collected, including the organ manifestation based on microbiological and radiological detection. Results of microbiological detection methods (*Mtb* culture, microscopy, polymerase chain reaction (PCR)) in extrapulmonary samples leading to diagnosis were recorded. The number of necessary invasive diagnostic procedures to obtain extrapulmonary samples for the pathogen detection was documented. For each patient, three sputum samples were tested for *Mtb* by culture, microscopy, and PCR. Patient data, including country of origin and refugee status, were collected. Based on other sources, we defined ‘refugees’ as those who made an involuntary choice to leave their country of origin [7, 13]. The region of origin was classified according to the World Bank definitions: East Asia and Pacific, Europe and Central Asia, Latin America and Caribbean, Middle East and North Africa, North America, South Asia, and Sub-Saharan Africa [14]. The category ‘sex’ was recorded as indicated by the patients themselves.

### Categories of disease severity

To date, there is no established staging system for EPTB to classify the disease extent and provide implications for treatment duration. In the pursuit of establishing such a system, patients in our cohort were classified into three distinct groups based on organ involvement. The category ‘disseminated TB’ was defined by the involvement of two or more non-contiguous organs, following widely accepted definitions that consider dissemination as the involvement of two or more non-contiguous organs, bloodstream, bone marrow, or liver [7]. We defined ‘mild disease’ as the isolated manifestation of TB in cervical lymph nodes, since only a very localized region was affected, while all other cases in between were classified as ‘intermediate’ stage.

### Paradoxical reactions

PR were defined as a worsening of TB lesions or the occurrence of new lesions based on clinical or radiological findings, following an initial improvement under effective treatment. Treatment failure, including drug resistance or non-compliance, needed to be excluded as a factor [15, 16].

### Clinical assessment of treatment response

Treatment response in our cohort was assessed through clinical and radiological findings, which guided the duration of treatment. Patients were monitored for one year post-treatment to check for relapse [17]. We also retrospectively evaluated the treatment response using a modified version of the scoring system proposed by Jorstad et al. After two months of therapy, Jorstad et al. applied a straightforward scoring system in a cohort of EPTB patients in Zanzibar, Tanzania, assigning 1 point each for a reduction in reported symptoms, a minimum 5% weight gain from baseline, and regression of objective findings, such as a decrease in lymph node size observed clinically [12]. We adjusted the parameters to match our diagnostic capabilities, particularly for the ‘regression of objective findings’ category. Instead of using visual estimates or assumptions, we systematically assessed physical sign regression through imaging. Routine imaging results were categorized as progression, regression, stable, or mixed, compared to the most recent prior scan. Patients were asked about pre-defined symptoms, and body weight was measured at baseline and follow-up. Points were assigned for symptom improvement, weight gain of ≥ 5%, and regression in imaging findings, following Jorstad et al.‘s methodology [12]. We assessed treatment response after 3 months. Proportions of patients within each cohort having a score of 0–3 points were calculated and compared.

### Statistical methods

Patient characteristics were presented using absolute numbers, percentages, medians, ranges, and interquartile ranges (IQR) as appropriate. If data on a certain aspect were not available for all patients, the examined sample size *n* was named. Non-parametric statistical methods were employed. The Mann-Whitney U test was utilized to compare two groups, while the Kruskal-Wallis test was applied for comparisons involving more than two groups. Post-hoc analysis after Kruskal-Wallis test was performed with Dunn’s multiple comparison test. Chi-square and Fisher’s exact tests were conducted for contingency analysis. A *p*-value of 0.05 was considered statistically significant. Statistical analysis and figure generation were performed using GraphPad Prism Version 10.2.2.

## Results

Between August 2018 and August 2023, a total of 44 patients were enrolled. Treatment was completed by 39/44 (89%) while 5/44 (11%) were lost to follow-up.

### Characteristics of the EX-TB cohort

**Fig. 1:**
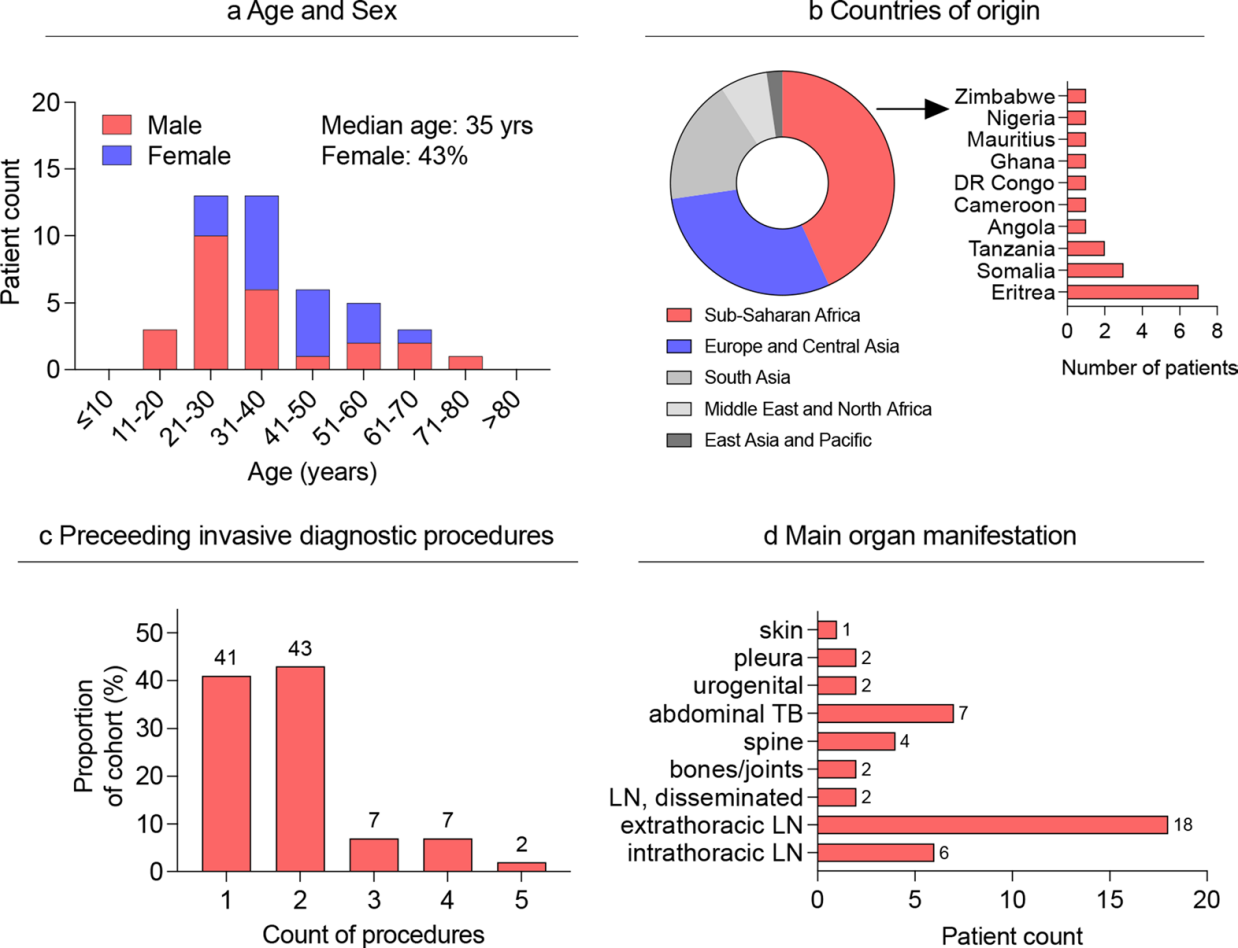
**a**) Distribution of age and sex of the participants. **b**) Pie chart depicting the origin of patients by regions according to the World Bank. Bar chart illustrating the distribution of countries of origin from Sub-Saharan Africa. **c**) Bar chart illustrating the proportion of invasive diagnostic procedures before pathogen detection. **d**) Bar chart depicting the main organ manifestation. TB, tuberculosis; LN, lymph node

The 44 patients in the cohort had a median age of 35 (range 18 to 80) years at the time of diagnosis (Fig. 1a). They originated from 21 different countries, most commonly Germany (16%, 7/44), and Eritrea (16%, 7/44, Fig. 1b). A history of refugee status was reported by 41% (18/44). Human immunodeficiency virus (HIV) coinfections were observed in 7% (3/44). The other most common comorbidity was arterial hypertension in 7% (3/44). When categorized according to the three disease severity categories defined in the methods section, 34% (15/44) were classified as having mild disease, 23% (10/44) as intermediate, and 43% (19/44) as disseminated disease. Drug resistance occurred in 11% of cases (5/44), including 4 cases of monoresistance to isoniazid and one case of multidrug resistance (Table 1).

**Table 1:** Patient characteristics of the Cologne EX-TB cohort (*n* = 44)

Median age	35 (range 18 to 80)
Sex female / male / non-binary	19/44 (43%) / 25/44 (57%) / 0/44 (0%)
Refugee	18/44 (41%)
Main Organ manifestation	
- Lymphatic	26/44 (59%)
- Bones/joints/spine	6/44 (14%)
- Abdominal	7/44 (16%)
- Urogenital	2/44 (5%)
- Pleural	2/44 (5%)
- Skin	1/44 (2%)
Comorbidities	
- HIV	3/44 (7%)
- Arterial hypertension	3/44 (7%)
- Diabetes mellitus	2/44 (5%)
- Renal insufficiency requiring dialysis	1/44 (2%)
- Syphilis	2/44 (5%)
- Others	23/44 (52%)
- No comorbidity	19/44 (43%)
Median time symptoms to diagnosis (months)	4 (range 1 to 23)
Drug sensitivity	
- Pan-susceptible pathogen	36/44 (82%)
- Isoniazid mono-resistance	4/44 (9%)
- Multi drug resistance	1/44 (2%)
- Unknown	3/44 (7%)
Positive sputum culture	8/44 (18%)
- Without pulmonary involvement in imaging	5/8 (63%)
Currently finished treatment	39/44 (89%)
- Median treatment duration (months)	10
- Lost to follow-up	5/44 (11%)
Study visit one year after treatment stop	30/44 (68%)
- Relapse	0/30 (0%)

The majority of patients (59%, 26/44) underwent two or more invasive procedures before receiving a confirmed diagnosis (Fig. 1c and supplementary Table 1). The median time between symptom onset and treatment initiation was 4 months (range: 1–23 months). In the extrapulmonary samples collected for diagnosis, microscopy yielded positive results in 20% (9/44), PCR gave positive results in 73% (32/44), positive cultures confirmed the diagnosis in 89% (39/44, supplementary Table 1). Microbiological detection in extrapulmonary samples was not successful in 7% (3/44) of the patients (supplementary Table 1), but one of these patients had a positive sputum culture. Sputum samples from all patients were examined, and sputum culture yielded positive results in 18% (8/44), with 75% of these (6/8) also showing positive results on sputum PCR. Among the 8 patients with positive sputum culture results, 63% (5/8) did not exhibit any radiological evidence of pulmonary involvement based on CT (3/5) or X-ray (2/5). According to the inclusion criteria, extrapulmonary manifestations were clinically dominant in all these patients.

### Paradoxical reactions (PR)

**Fig. 2:**
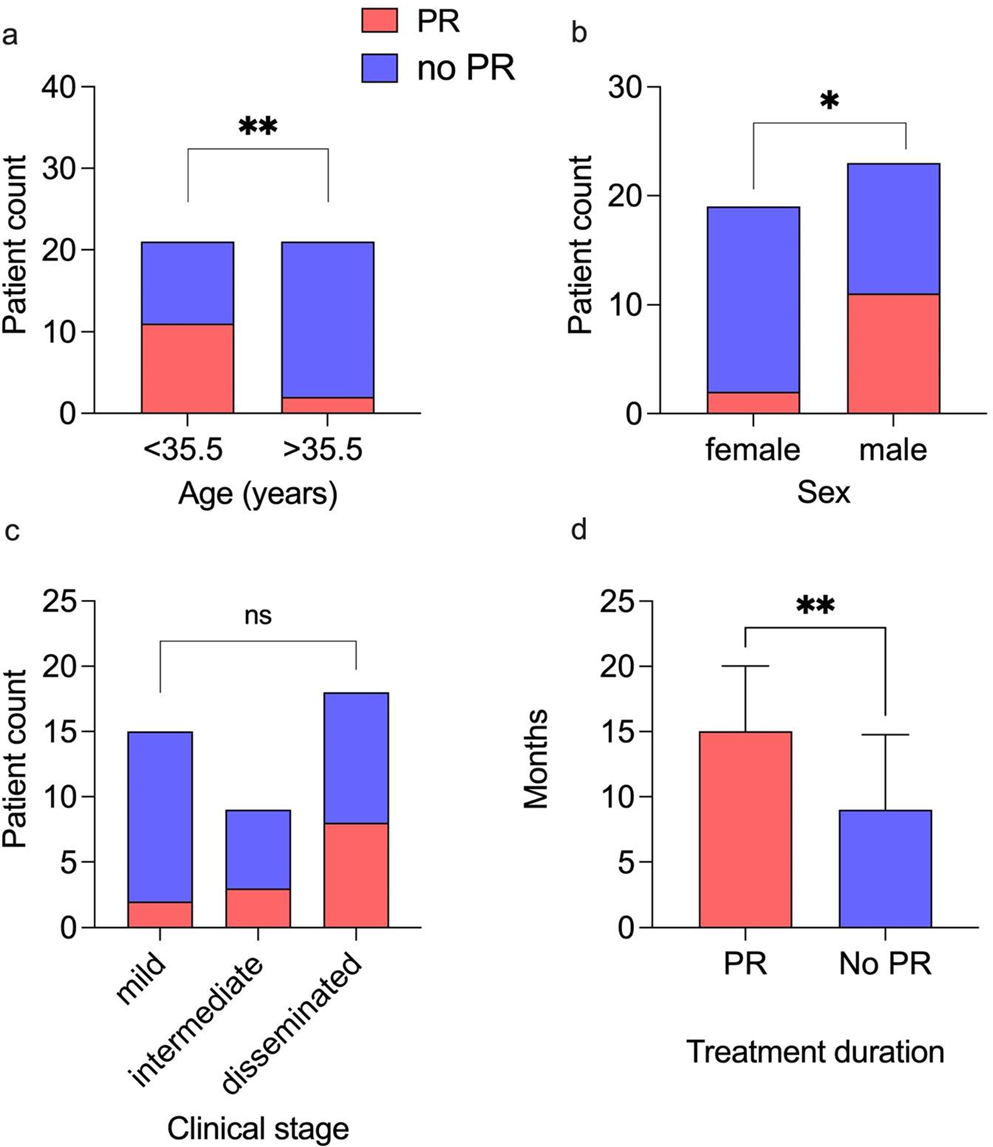
Distribution of patients without (blue) and with (red) paradoxical reactions (PR) during the course of treatment, subdivided by median age (**a**), sex (**b**) and clinical stage (**c**). Treatment duration of patients with PR vs. patients without PR (**d**). PR, paradoxical reaction

PR occurred in 31% (13/42) of the patients who were followed up. Clinical signs of suspected PR were most commonly pain (31%, 4/13) and swelling of palpable lesions (31%, 4/13). Fever occurred in 8% (1/13). Correlates in imaging were found in 82% (9/11). Steroids were applied in 69% (9/13), an invasive procedure was performed in 15% (2/13). In 31% (4/13) of patients with PR, the condition resolved without specific therapy (see supplementary Table 2). PR occurred after a median treatment duration of 2 months (range 0 to 8 months). The median age of patients with PR was 28 years (ranging from 21 to 50), whereas patients without PR had a median age of 40 years (ranging from 18 to 80). The proportion of PR in patients younger than the overall median age was significantly higher compared to those older than the median age (*p* = 0.0063). The proportion of PR in female patients was significantly lower than in male patients (*p* = 0.0173). The proportion of PR did not differ among patients within the different clinical stages (*p* = 0.1544). Treatment duration in patients with a PR was significantly longer (median 15 months) compared to patients without PR (median 9 months), (*n* = 39, *p* = 0.0038) (Fig. 2).

### Clinical evaluation of treatment response

**Fig. 3:**
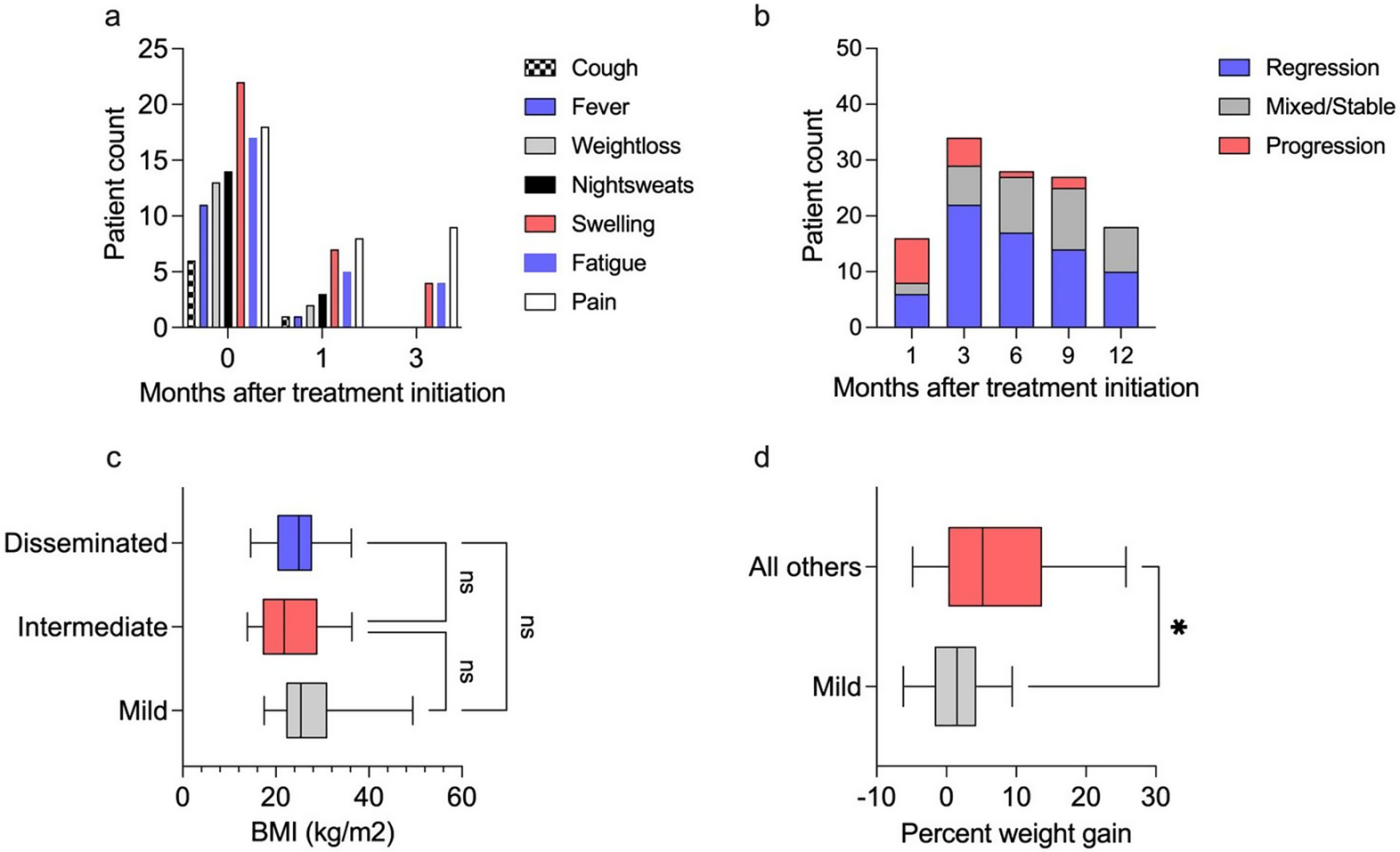
**(a)** Bar chart depicting the number of patients reporting the various symptoms at the start of treatment, and at 1 and 3 months after initiation of therapy, respectively. **(b)** Bar chart depicting the number of patients having received imaging through the course of treatment, divided by changes in imaging towards the last performed imaging before: regressive findings (blue), mixed/stable findings (gray), and progressive findings (red). **(c)** Boxplot depicting Body Mass Index (BMI) in kg/m2 at baseline in patients with disseminated disease stage (blue), intermediate disease stage (red) and mild disease stage (gray) before treatment initiation (median, interquartile ranges and minimum and maximum values), *n* = 36. **(d)** Boxplot depicting percent weight gain after six months of treatment compared to baseline weight in patients with mild disease (gray), compared to all other patients (red) (median, interquartile ranges and minimum and maximum values), *n* = 39

The most commonly reported symptom at baseline was swelling at the affected organ site, observed in 52% (23/44), followed by pain in 41% (18/44) and fatigue in 39% (17/44, Fig. 3a). One month after treatment initiation, 81% (35/43) reported a reduction in symptoms. The most frequently reported symptoms after one month of treatment were pain (19%, 8/43), and swelling (16%, 7/43). Three months after treatment initiation, the most frequent symptom was pain reported by 21% (9/43). An increase in the numbers of reported symptoms at the 3-months study visit was observed in 16% (7/43), of whom 2 had PR (29%, 2/7). Imaging data showed a 50% rate (8/16) of progressive disease after 1 month of treatment. In contrast, after 3 months of treatment, regressive findings in imaging were most common (65%, 22/34), as well as during the later course of treatment (Fig. 3b).

Baseline BMI showed no differences across the various disease stages. The percent of weight gained after 6 months of treatment was significantly lower in patients with mild disease compared to those in other stages (*p* = 0.0221, Fig. 3c and d).

### Adapted treatment response score by Jorstad et al

**Fig. 4:**
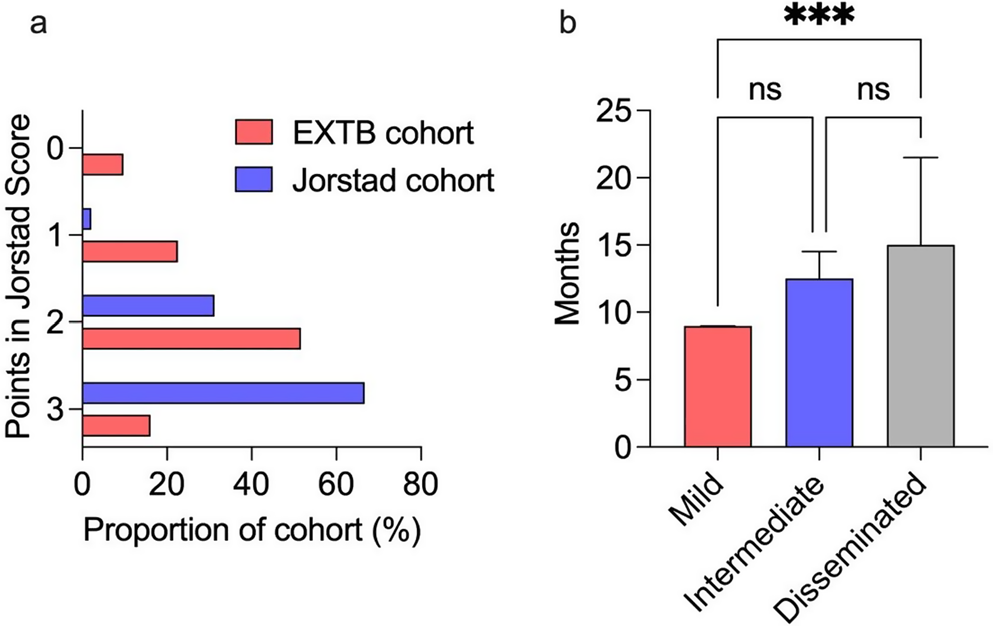
**a)** Bar chart depicting the proportions of patients in both cohorts having in sum 0 to 3 clinical parameters after 3 months of treatment in EX-TB cohort, and 2 months in Jorstad cohort. Clinical parameters were given each one point for (**a**) reduction of symptoms, (**b** weight gain > 5% compared to baseline and (**c**) regressive findings in objective findings (imaging or examination). Cologne EX-TB cohort *n* = 31 (red), cohort by Jorstad et al. *n* = 48 (blue). **b)** Bar chart depicting the treatment duration in months, subdivided by mild disease (red), intermediate disease (blue) and disseminated disease (grey). Shown values are median with interquartile ranges, *n* = 39

To better assess treatment responses we also applied the adapted score of Jorstad et al. which is based on three clinical categories ‘symptom reduction’, ‘weight gain > 5%’ and ‘regression in objective findings’, e.g. lymph node size [12]. Data were available for 31 patients after 3 months of treatment. If data were unavailable, weight and imaging data were used from an earlier timepoint, which was the case for 2 patients regarding imaging data and 2 patients regarding weight changes. Overall, a score of 2 or more points was achieved by 68% (21/31) of the patients (Fig. 4a), compared to 98% (47/48) attaining a score of ≥ 2 points in the cohort of Jorstad et al. [12]. A major difference between our cohort and that of Jorstad et al. was primarily observed in the category ‘weight gain’ (weight gain of ≥ 5% in 73% (36/49) after two months of treatment vs. only 29% (9/31) in our cohort even after 3 months). It is worth noting that, in this cohort, the lower percentage of patients with a score of 2 points or more did not indicate treatment failure. After one year of follow-up, none of the 30 monitored patients (out of 44) experienced a relapse.

### Treatment duration

To date, 89% (39/44) of the patients have completed treatment, with a median total treatment duration of 10 months (range 6 to 37), all others were lost to follow-up (11%, 5/44). When stratified by disease severity, the median treatment duration was 9 months (range 6 to 18) for patients with mild disease, 13 months (range 9 to 20) for those with intermediate disease, and 15 months (range 9 to 37) for patients with disseminated disease (Fig. 4b). Notably, the 18-month treatment duration in one patient with mild disease was attributed to MDR TB. In another case, a 37-month treatment duration was required due to disseminated bone lesions with persistent changes observed in imaging. Treatment durations between the three groups differed significantly (*p* = 0.0009), although in a post-hoc analysis a significant difference was only seen between mild and disseminated disease (adjusted *p* = 0.0006) (Fig. 4b). A documented follow-up visit one year after the end of the study was conducted in 68% (30/44) of the patients, with no radiological or clinically confirmed relapses recorded.

## Discussion

This cohort study depicts the clinical heterogeneity of EPTB in a low incidence setting. Repeated diagnostic sampling is often necessary for pathogen detection, resulting in delayed treatment initiation. A high rate of PR, particularly in younger patients, was observed. Treatment durations varied significantly across the three stages of disease extent, highlighting the need for disease stratification. A clinical scoring system for assessing treatment response that proved useful elsewhere [12] was effective in only two-thirds of the patients.

The cohort also demonstrated considerable diversity in organ manifestations, disease extent, and geographic origins. Aside from Germany, Eritrea was the most common country of origin. A German study found Eritrean origin was significantly linked to EPTB over PTB [18].

The month-long delay from symptom onset to diagnosis and treatment initiation we observed aligns with Mathiasen et al.‘s systematic review, which reported a median diagnostic delay of 77.5 to 122 days for lymph node TB in low-incidence settings [19]. Physicians’ lack of awareness of EPTB in countries with low TB incidence, combined with nonspecific symptoms, can contribute to diagnostic challenges [19]. The diagnostic latency carries significant consequences, as delayed initiation of treatment is associated with increased mortality rates and greater disease severity, evident in both PTB and EPTB [20, 21]. Additionally, the need for invasive sampling in EPTB can further prolong the time until diagnosis. The majority of the patients in our cohort underwent two or more invasive diagnostic procedures, such as biopsies or lymph node extirpations, eventually leading to pathogen detection. In low TB-incidence settings, enlarged lymph nodes or other organ lesions are often attributed to malignancies rather than infections. Consequently, material obtained from biopsies is commonly preserved in formalin, rendering it unsuitable for subsequent microbiological tests such as culture and molecular diagnostics. This necessitates multiple invasive procedures to obtain native samples allowing for successful bacterial cultures and molecular diagnostics. The relatively high number of patients with positive sputum results highlights the significance of sputum diagnostics in EPTB, even in those without radiological signs of lung involvement.

Paradoxical reactions (PR) are frequently described in EPTB [22, 23] and could potentially delay a patient’s recovery. HIV-infection is a well-known risk for PR due to immune reconstitution after initiation of antiretroviral treatment [24]. Although only a small percentage of patients in our cohort were HIV-positive (7%, 3/44), PR occurred in 31% of cases. This rate is relatively high but consistent with other studies, reporting PR rates in HIV-negative EPTB patients ranging from 15 to 35% [15, 24, 25, 26]. The underlying mechanism for PR in HIV-negative patients remains poorly understood [23, 28]. However, several risk factors for PR in EPTB without HIV-infection have been discussed, e.g. manifestation of TB in multiple sites, and extrapulmonary manifestation itself, potentially indicating a higher antigen load [24, 25], which promotes inflammatory responses. In our cohort, PR occurred more often in patients with disseminated TB than in other disease stages, though this was not statistically significant. The frequency of PR was significantly lower among older patients, a trend that has been observed elsewhere [25, 27, 29]. This is attributed to the impairment of T-cell-modulated immune responses with age [30, 31]. Furthermore, in our cohort, male sex was associated with the occurrence of PR, a finding also observed in other studies [29]. In our study, patients with PR were treated longer with antimicrobial therapy than those without PR, indicating the need for a better understanding of PR to avoid treatment extensions which may be unnecessary.

The World Health Organization (WHO) recommends a 6-months treatment for drug-susceptible EPTB, extending to 9–12 months for cases involving the central nervous system, bones, or joints [2]. Treatment duration for EPTB should be guided by clinical findings and imaging dynamics [32, 33]. However, in our cohort, imaging frequently revealed progressing TB lesions even after 9 months of treatment which did not correlate with treatment failure. Therefore, the role of imaging in evaluating treatment response in EPTB needs careful consideration. Recent studies suggest that advanced imaging techniques, such as multimodal ultrasound for tuberculous lymphadenitis and 18 F-FDG PET/CT for both PTB and EPTB, may be more valuable than traditional methods like X-ray, basic ultrasound, MRI, and CT [34, 35]. However, these techniques are unlikely to distinguish between treatment failure and PR.

In need of reliable tools to monitor treatment response in EPTB, we applied the clinical treatment response score initially proposed by Jorstad et al. [12]. This score was successfully tested in a cohort of EPTB patients in Zanzibar, Tanzania, where 98% demonstrated a positive treatment response. In our cohort, only two-thirds demonstrated a similar pattern leaving more than 30% of patients with a false negative result, primarily due to a lack of weight gain. Our study was conducted in a high-resource, low-incidence setting. Studies in which weight gain demonstrated its potential as a predictor for treatment outcome in certain settings were conducted in socioeconomically deprived environments with high rates of undernourishment [36, 37]. In contrast, in our specific context, weight gain did not prove to be a reliable indicator of treatment response. In conclusion, the adapted score proposed by Jorstad et al. may perform better in high-incidence than low-incidence settings.

We suggest stratifying EPTB using a clinical staging system that distinguishes between isolated cervical lymph node TB, disseminated TB, and an ‘intermediate’ stage encompassing all other forms. In our cohort, treatment duration varied significantly across these stages. The extremely low rate of treatment failure observed highlights the clinical relevance of this approach. Patients with more severe organ manifestations, such as bone, spine, or joint involvement, received longer treatment than recommended by the WHO [38-40]. Thus, systematic ‘staging’ protocols, similar to those used in malignancies, may guide decisions on treatment length. Such an approach would involve baseline assessments of the involved body sites through imaging and tailoring treatment strategies to individual EPTB cases, thereby supporting clinicians in their decisions. However, this approach requires evaluation in prospective clinical trials.

Finally, it clearly appears that there is a need for valid, easily obtainable biomarkers helping to individualize treatment duration. Promising omics-based blood based biomarkers currently tested in clinical studies may predict suitable durations of EPTB therapy on an individual basis [41].

## Conclusion

We present a longitudinal cohort of EPTB patients in a low-incidence setting, characterized by significant heterogeneity in terms of geographical origin, refugee status, organ involvement, and disease severity. The diagnostic process is notably complicated by the requirement for invasive procedures to detect *Mtb* in EPTB, resulting in delayed treatment initiation. Clinical management is complicated by the high incidence of PR, particularly among younger and male patients. Assessing treatment response through a score of clinical parameters is feasible only for a limited number of patients, underscoring the urgent need for reliable biomarkers to aid in diagnostics and therapy monitoring. A clinical staging system to predict treatment duration based on disease severity may be valuable.

## Electronic supplementary material

Below is the link to the electronic supplementary material.

Supplementary Material 1

Supplementary Material 2

## Data Availability

No datasets were generated or analysed during the current study.
