# Supplementary material for: Clinical heterogeneity and treatment outcomes of extrapulmonary tuberculosis in a low-incidence setting: insights from a prospective cohort study
Source: Infection. 2025 Mar 11;53(5):1809–18. doi: 10.1007/s15010-025-02500-4 (PMC12460547; doi:10.1007/s15010-025-02500-4)
Supplement: Supplementary file 2 — Supplementary Material 2 [file 15010_2025_2500_MOESM2_ESM.docx]

**Supplements**

**Supplementary tables**

|  | Total number of interventions | Performed interventions | Extrapulmonary sample leading to diagnosis | PCR | Microscopy | Culture | | Sputum  (PCR/microscopy/culture) |  |
| --- | --- | --- | --- | --- | --- | --- | --- | --- | --- |
| Patient 1 | 1 | lymph node exstirpation | lymph node | + | - | + | - | |  |
| Patient 2 | 2 | fine needle punction; lymph node exstirpation | lymph node | + | - | + | - | |  |
| Patient 3 | 2 | lymph node exstirpation | lymph node | + | - | + | - | |  |
| Patient 4 | 2 | fine needle punction; lymph node exstirpation | lymph node | - | - | - | - | |  |
| Patient 5 | 1 | sternal punction | lymph node | + | - | + | - | |  |
| Patient 6 | 2 | wedge resection; lymph node punction | lymph node | + | - | + | +/-/+ | |  |
| Patient 7 | 2 | bronchoalveolar lavages | no extrapulmonary sample obtained | - | - | - | +/-/+ | |  |
| Patient 8 | 2 | spinal punction; spinal surgery | bone | + | - | + | - | |  |
| Patient 9 | 1 | subfascial biopsy | bone | + | - | + | +/-/+ | |  |
| Patient 10 | 1 | liver punction | liver | + | - | + | - | |  |
| Patient 11 | 4 | two laparotomies; two ascites punctions | lymph node | + | - | + | - | |  |
| Patient 12 | 2 | two lymph node exstirpations | lymph node | + | - | + | - | |  |
| Patient 13 | 5 | two endoscopic biopsies; transbronchial biopsy; two lymph node exstirpations | lymph node | - | - | + | - | |  |
| Patient 14 | 1 | lymph node punction | lymph node | + | - | + | - | |  |
| Patient 15 | 1 | abscess punction | bone | + | + | + | - | |  |
| Patient 16 | 2 | two lymph node exstirpations | lymph node | + | - | + | - | |  |
| Patient 17 | 1 | pleural biopsy | pleura | + | + | + | -/-/+ | |  |
| Patient 18 | 1 | endobronchial ultrasound guided lymph node punction | lymph node | - | - | + | - | |  |
| Patient 19 | 2 | lymph node punction; lymph node exstirpation | lymph node | + | + | + | - | |  |
| Patient 20 | 3 | cervical lymph node exstirpation; lymph node punction; laparoscopic lymph node exstirpation | lymph node | + | - | + | - | |  |
| Patient 21 | 2 | testicle biopsy; ablatio testis | testicle | - | - | - | - | |  |
| Patient 22 | 4 | two ascites punctions; CT-guided punction; minilaparoscopy | peritoneum | + | - | + | - | |  |
| Patient 23 | 3 | CT-guided mediastinal lymph node punction; VATS-assisted lymph node biopsy; lymph node exstirpation | lymph node | + | - | + | - | |  |
| Patient 24 | 2 | pleural punction; VATS-assisted pleural biopsy | pleura | + | + | + | - | |  |
| Patient 25 | 2 | lymph node exstirpation; lymph node biopsy | lymph node | - | + | - | - | |  |
| Patient 26 | 2 | lymph node exstirpations | lymph node | - | - | + | - | |  |
| Patient 27 | 1 | CT-guided lymph node punction | lymph node | + | - | + | - | |  |
| Patient 28 | 3 | three lymph node exstirpations | lymph node | + | - | + | - | |  |
| Patient 29 | 1 | thyroid biopsy | lymph node | + | - | + | -/-/+ | |  |
| Patient 30 | 2 | skin biopsy; lymph node exstirpation | lymph node | + | - | + | - | |  |
| Patient 31 | 2 | two knee operations | bone | - | - | + | - | |  |
| Patient 32 | 1 | lymph node wedge resection | lymph node | + | - | + | - | |  |
| Patient 33 | 1 | Lymph node wedge resection | lymph node | - | - | + | - | |  |
| Patient 34 | 2 | fine needle punciton; lymph node exstirpation | lymph node | + | - | + | - | |  |
| Patient 35 | 1 | lymph node biopsy | lymph node | + | + | + | +/+/+ | |  |
| Patient 36 | 1 | nephrectomy | kidney | + | + | + | - | |  |
| Patient 37 | 2 | lymph node exstirpations | lymph node | - | - | + | - | |  |
| Patient 38 | 2 | lymph node exstirpations | lymph node | - | - | + | - | |  |
| Patient 39 | 1 | skin biopsy | skin | + | - | - | -- | |  |
| Patient 40 | 1 | spinal surgery | bone | + | - | + | +/-/+ | |  |
| Patient 41 | 4 | three ascites punctions; laparoscopic biopsy | ascites | - | - | + | - | |  |
| Patient 42 | 1 | pancreatic surgery | pancreas | + | - | + | - | |  |
| Patient 43 | 1 | spinal biopsy | bone | + | + | + | +/-/+ | |  |
| Patient 44 | 2 | lymph node exstirpations | lymph node | + | + | + | - | |  |

**Supplementary Table 1:** Table depicting the number and kinds of necessary invasives procedures for diagnosis of extrapulmonary tuberculosis and microbiological pathogen detection in extrapulmonary and pulmonary (n=44)

| Symptoms |  |
| --- | --- |
| - Pain | 4/13 (31%) |
| - Swelling | 4/13 (31%) |
| - Fever | 1/13 (8%) |
| - Fistula | 1/13 (8%) |
| - No Symptoms | 3/13 (23%) |
| Imaging |  |
| - Performed | 11/13 (85%) |
| - Correlate for PR in imaging | 9/11 (82%) |
| Treatment |  |
| - Corticoids | 9/13 (69%) |
| - Intervention | 2/13 (15%) |
| - Spontaneous remission | 4/13 (31%) |
| Median time of occurrence of PR after treatment starts (months) | 2 (range 0 to 8) |

**Supplementary Table 2:** Characteristics of paradoxical reactions
